# Influenza A(H3N2) Subclade K (J.2.4.1): Molecular Characterization, Antigenic Divergence, and Global Spread During the 2025/26 Season

**DOI:** 10.3390/idr18020037

**Published:** 2026-04-14

**Authors:** Francesco Branda, Nicola Petrosillo, Giancarlo Ceccarelli, Fabio Scarpa, Marta Giovanetti, Massimo Ciccozzi

**Affiliations:** 1 Unit of Medical Statistics and Molecular Epidemiology, Università Campus Bio-Medico di Roma, 00128 Rome, Italy; m.ciccozzi@unicampus.it; 2 Infection Prevention & Control/Infectious Disease Service, Fondazione Policlinico Universitario Campus Bio-Medico, 00127 Rome, Italy; 3 Department of Public Health and Infectious Diseases, University Hospital Policlinico Umberto I, Sapienza University of Rome, 00185 Rome, Italy; giancarlo.ceccarelli@uniroma1.it; 4 Department of Biomedical Sciences, University of Sassari, 07100 Sassari, Italy; fscarpa@uniss.it; 5 Department of Science and Bio-Technology, Università Campus Bio-Medico di Roma, 00128 Rome, Italy; giovanetti.marta@gmail.com; 6 Instituto René Rachou, Fundação Oswaldo Cruz, Belo Horizonte 30190-002, Brazil; 7 Climate Amplified Diseases and Epidemics (CLIMADE) Americas, Belo Horizonte 30190-002, Brazil

**Keywords:** influenza A(H3N2), subclade K, J.2.4.1, antigenic drift, hemagglutinin (HA), neuraminidase (NA), vaccine effectiveness, phylogenetic analysis, global surveillance, seasonal influenza

## Abstract

**Background:** Influenza A(H3N2) continues to evolve rapidly, frequently eroding population immunity and challenging seasonal vaccine strain selection. During the 2025/26 season, the A(H3N2) subclade K (J.2.4.1) expanded quickly across multiple regions and showed evidence of antigenic divergence in standard assays. **Methods:** In this study, we combined phylogenetic analyses of hemagglutinin (HA) and neuraminidase (NA) sequences with a systematic synthesis of recent peer-reviewed studies and official surveillance reports to comprehensively define the molecular profile and early epidemiological dynamics of subclade K. **Results:** Our phylogenetic reconstructions of HA and NA genes confirmed the emergence of a coherent and recently diversified lineage characterized by coordinated evolution of surface glycoproteins and broad geographic representation during 2025. Integration of molecular, temporal, and surveillance evidence further supported rapid expansion with limited early regional structuring. Antigenic analyses reported in peer-reviewed studies described reduced haemagglutination inhibition reactivity to vaccine reference antisera for many subclade K viruses, whereas vaccine effectiveness (VE) estimates from multiple settings remained moderate. **Conclusions:** Overall, the available genetic, antigenic, and epidemiological evidence indicates that subclade K represents a recently diversified A(H3N2) lineage associated with rapid international spread during the 2025/26 season, highlighting the importance of integrated HA/NA genomic surveillance and timely antigenic characterization to support evidence-based vaccine strain selection.

## 1. Introduction

Since its pandemic emergence in 1968, influenza A(H3N2) has remained one of the most persistent and rapidly evolving respiratory pathogens in humans, driving annual seasonal epidemics and disproportionately affecting older adults and individuals with comorbidities. Compared with A(H1N1)pdm09 and influenza B viruses, H3N2 typically evolves more rapidly at both the genetic and antigenic levels, increasing the likelihood of mismatch between vaccine components and circulating viruses and contributing to seasons with substantial healthcare burden [1].

This continual turnover is underpinned by selective pressure on the viral surface glycoproteins hemagglutinin (HA) and neuraminidase (NA), which must jointly preserve efficient entry and release while evading population immunity [1]. Most neutralizing antibodies target epitopes on the HA head near the receptor-binding site (RBS), a region that accumulates substitutions and glycosylation changes that reduce recognition while maintaining receptor engagement. Accordingly, antigenic drift is often realized through a constrained set of recurrent solutions, including remodeling of surface loops, shifts in charge, and gains or losses of N-linked glycosylation. Historical examples include clade-defining HA mutations such as F159Y and K160T, with the latter enabling addition of a glycan that contributed to antigenically distinct clades and reduced vaccine performance in the mid-2010s [1]. Contemporary H3N2 viruses also pose practical surveillance challenges, as changes in receptor usage and glycosylation can affect virus propagation and the readout of classical antigenic assays [2].

Vaccine strain selection is further complicated by the propagation of candidate vaccine viruses, particularly in egg-based manufacturing, where adaptive substitutions can be selected near the HA RBS and can alter antigenicity relative to the intended target virus [3]. In addition, antigenic change is not restricted to HA: NA-specific antibodies can contribute to protection, and NA evolution, including glycosylation changes near functional sites, can modulate immune recognition and complement HA-mediated drift [4]. At the same time, H3N2 fitness depends on coordinated, genome-wide compatibility; for example, HA changes that affect receptor avidity may require compensatory NA changes, and internal-gene substitutions can help maintain replication competence under immune pressure [5].

During the 2025/26 season, the rapid global rise of influenza A(H3N2) subclade K (J.2.4.1) provided a timely example of these dynamics. Characterization studies reported reduced haemagglutination inhibition (HI) reactivity of many subclade K isolates relative to contemporary vaccine reference viruses, consistent with antigenic displacement in standard assays [6,7,8]. Despite these laboratory drift signals, interim vaccine-effectiveness estimates from England, Canada, and Beijing suggested that protection against medically attended influenza remained measurable during early subclade K predominance [7,9,10]. At the strain-selection level, World Health Organization (WHO) vaccine composition recommendations for the 2025 southern hemisphere and 2025/26 northern hemisphere seasons, together with complementary summaries from the Centers for Disease Control and Prevention (CDC), the Food and Drug Administration (FDA), the European Medicines Agency (EMA), and the European Centre for Disease Prevention and Control (ECDC), synthesized multi-country genetic and antigenic evidence and provided additional context for interpreting assay findings and uncertainties [11,12,13,14,15,16].

Recent global influenza surveillance outputs have further informed the most recent WHO recommendations for the composition of influenza vaccines for the 2026/27 Northern Hemisphere season, as stated in the WHO Weekly Epidemiological Record (WER) [17]. These recommendations were derived from the integrated analysis conducted within the Global Influenza Surveillance and Response System (GISRS), incorporating virological, antigenic, and epidemiological evidence from multiple regions, including data reported through FluNet, national influenza centers, and sequence repositories such as Global Initiative on Sharing All Influenza Data (GISAID). A central element of the WHO consultation was the continued expansion and global predominance of A(H3N2) viruses within the 2a.3a.1 genetic group, and in particular subclade J.2.4.1 (subclade K), according to the nomenclature system proposed by Neher et al. [18], which exhibited widespread circulation across both hemispheres during the 2025/26 season. Antigenic characterization data summarized in the WER report indicated measurable reductions in haemagglutination inhibition reactivity of J.2.4.1 viruses relative to the previously recommended vaccine reference strain, consistent with antigenic drift at key HA epitopes. Importantly, these findings were interpreted in conjunction with genetic data and early epidemiological indicators, highlighting the multifactorial nature of vaccine strain selection. The WHO recommendation process emphasized that vaccine composition decisions cannot rely solely on antigenic distance estimates from ferret antisera, but require an integrated assessment of HA and NA evolution, global transmission dynamics, and interim vaccine effectiveness (VE) data. In this context, the rapid international spread and antigenic displacement of subclade K provided convergent evidence supporting its relevance in shaping the 2026/27 vaccine update. This further underscores the importance of real-time genomic surveillance and coordinated global data sharing for anticipating antigenic evolution in rapidly drifting influenza A(H3N2) viruses.

Interpretation of these interim VE estimates requires consideration of study context, population factors, and temporal dynamics. VE commonly differs by endpoint (medically attended outpatient illness versus hospitalization), age and prior exposure history, vaccine platform and product mix, and the timing of evaluation within the season (including potential within-season waning). Recent test-negative studies conducted during early subclade K predominance reported moderate VE in outpatient settings, including an adjusted VE of 41.3% (95% CI: 29.2–51.3) in Beijing (September–December 2025) [10] and interim VE estimates of approximately 40% against medically attended illness due to predominant A(H3N2) viruses (including subclade K) in Canada (January 2026) [9]. Moreover, serologic reductions measured with ferret antisera in HI assays do not necessarily translate linearly to population-level VE, which reflects immune mechanisms beyond HI-measured antibodies, including NA-directed responses and non-neutralizing antibody effector functions [1].

This work investigates the emergence and expansion of subclade K using paired HA and NA phylogenetic analyses and a synthesis of publicly available epidemiological and surveillance evidence. Section 2 outlines the study design, data sources, and analytical approach. Section 3 presents the main results, describing the genetic features of subclade K, the integration of publicly available HA and NA genome sequences within a phylogenetic reconstruction framework to define the evolutionary relationships of subclade K viruses available antigenic evidence, and analysis of World Health Organization (WHO) FluNet time-series data to evaluate global circulation trends. Finally, Section 4 provides a comprehensive discussion of the implications for genomic surveillance and vaccine strain selection, delineates the principal limitations and remaining knowledge gaps, and defines priorities for the continued monitoring of H3N2 evolutionary trajectories.

## 2. Materials and Methods

This study combined genomic characterization of influenza A(H3N2) viruses with a synthesis of public surveillance evidence to describe the emergence and early spread of the A(H3N2) subclade K lineage and to contextualize its antigenic and epidemiological relevance. The primary objectives were: (i) to reconstruct the evolutionary history, temporal dynamics, and global genetic structure of subclade K through phylogenetic analyses of hemagglutinin (HA) and neuraminidase (NA) sequences; and (ii) to summarize contemporaneous surveillance and vaccine-effectiveness evidence from the 2025/26 season.

From an epidemiological perspective, data were obtained from the WHO FluNet platform (https://www.who.int/tools/flunet, accessed on 10 February 2026). The dataset consisted of aggregated time series describing global influenza activity and subtype circulation across countries. FluNet integrates heterogeneous surveillance inputs from multiple reporting systems, including sentinel, non-sentinel, and undefined streams. Sentinel surveillance refers to systematic sampling from predefined outpatient networks using standardized case definitions (e.g., influenza-like illness (ILI) or acute respiratory infection (ARI)), enabling consistent temporal comparisons over time. Non-sentinel surveillance generally reflects testing performed outside sentinel networks, including hospital-based testing, outbreak investigations, and routine diagnostic testing, and may be influenced by variations in healthcare-seeking behavior and testing intensity. Undefined (or unknown) streams include reports for which the surveillance source is not specified or cannot be clearly assigned to either sentinel or non-sentinel categories. FluNet data were analyzed as reported, without normalization for population size, testing volume, or surveillance coverage. Accordingly, the resulting time series should be interpreted as indicators of surveillance activity and temporal dynamics rather than direct estimates of incidence or disease burden. Geographic stratification followed the predefined regional categories available within the FluNet interface. Because data are aggregated at the national level, countries spanning multiple climatic or epidemiological zones are assigned to a single category, which may introduce ecological bias and limit the interpretation of regional comparisons. Influenza A detections reported without subtype specification were retained as a separate category to avoid introducing assumptions in subtype assignment. These records may reflect incomplete laboratory characterization or differences in reporting practices across surveillance systems.

From a phylogenetic perspective, analyses were performed using publicly available, de-identified sequence data and associated metadata, without access to individual-level human participant information, and therefore did not require additional ethical approval. Available H3N2 sequences were downloaded from GISAID by gene segment (HA and NA), applying filters for sequence completeness, quality, and availability of sampling metadata. To minimize geographic sampling bias, sequences were selected to ensure representative coverage across different regions. In total, 2040 sequences per gene were included in the analyses, together with 30 ancestral H3N2 3C.3a reference sequences used to provide phylogenetic context. Sequence alignments for the two surface antigen segments (HA and NA) were generated using ViralMSA with minimap2 [19,20]. Alignments were manually inspected and curated in AliView to remove potential biological inconsistencies and alignment artifacts, including frameshifts and terminal gaps [21]. Maximum-likelihood (ML) phylogenetic inference for each alignment was performed using IQ-TREE2 [22], employing the general time-reversible model of nucleotide substitution with a proportion of invariable sites (+I), as selected by ModelFinder. Time-scaled phylogenies were subsequently reconstructed using TreeTime only for sequences belonging to the K clade in each gene segment [23].

## 3. Results

### 3.1. Emergence and Phylogenetic Placement of Influenza A(H3N2) Subclade K (J.2.4.1)

**Figure 1:**
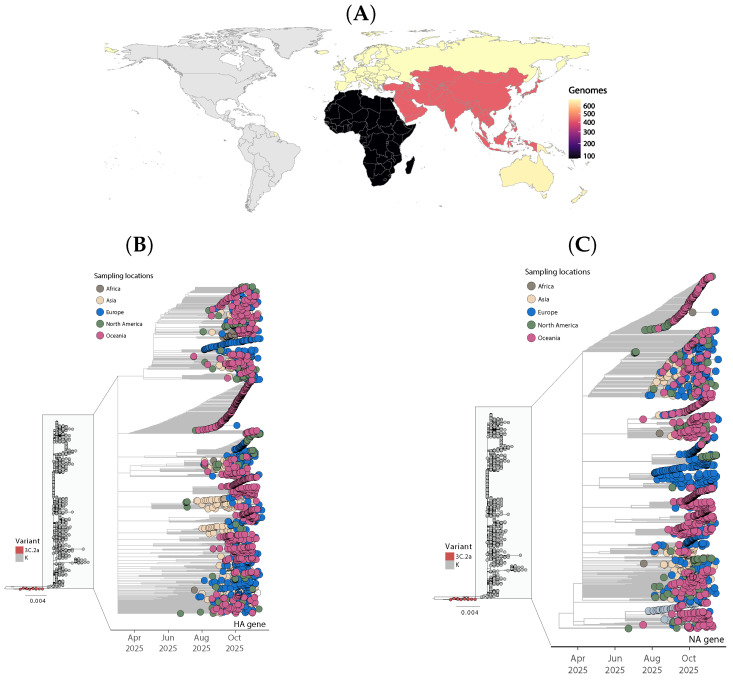
Global distribution and phylogenetic analysis of influenza A(H3N2) HA and NA genes. (**A**) Global distribution of H3N2 genomes included in this study. Countries are shaded according to aggregated genome counts derived from the study dataset and visualized using a predefined scaling scheme. (**B**,**C**) Time-scaled maximum-likelihood phylogenies of the HA (**B**) and NA (**C**) genes, including global reference sequences and subclade K viruses. Representative 3C.2a viruses were included for evolutionary context. Tips are colored by sampling location (Africa, Asia, Europe, North America, and Oceania), and clades are indicated in the inset panels (K in grey, 3C.2a in red). Branch lengths represent genetic divergence, and the x-axis indicates sampling time (2025). Both phylogenies show a well-supported K lineage within 3C.2a diversity, consistent with broad geographic dissemination.

To place the emergence of influenza A(H3N2) subclade K within its global and evolutionary context, we combined worldwide genome distribution data with time-resolved phylogenetic analyses of the HA and NA gene segments (Figure 1). These analyses revealed the recent emergence of a genetically distinct lineage within contemporary A(H3N2) diversity, corresponding to subclade K (J.2.4.1). Geographic patterns inferred from sampling metadata (Figure 1A) demonstrated broad representation of K viruses across multiple continents, including Europe, North America, Asia, Africa, and Oceania. The molecular signature of subclade K was detected across multiple continents, supporting rapid dissemination with limited time for extensive local diversification. In Beijing (September–December 2025), sequencing of a random subset of A(H3N2)-positive samples showed rapid predominance of subclade K (84.8% of 316 sequenced A(H3N2)-positive specimens) [10]. In northeast Ohio (autumn 2025), subclade K was identified through whole-genome sequencing, documenting early US circulation and reinforcing evidence of broad international seeding [6,24]. To place these molecular changes in a phylogenetic framework, we reconstructed maximum-likelihood phylogenies for the HA and NA segments; the HA tree (Figure 1B) shows the clustering of subclade K relative to other contemporaneous H3N2 lineages, whereas the NA tree (Figure 1C) demonstrates concordant clustering of subclade K-associated NA sequences, consistent with coordinated HA/NA evolution rather than a transient assortment of unrelated segment constellations [6]. In both HA and NA reconstructions, K viruses formed a well-supported monophyletic cluster nested within the broader 3C.2a genetic background, indicating recent divergence from previously circulating strains. The temporal structure of the phylogenies suggests that the K lineage expanded rapidly, with increasing representation in later sampling periods. Concordant clustering patterns observed across HA and NA segments support a coherent evolutionary trajectory rather than reassortment-driven emergence. The widespread distribution and intermixing of sequences from different regions within the same phylogenetic clusters are consistent with sustained international transmission and repeated cross-regional introductions during the 2025/26 season. No strong continent-specific substructure was observed within the K lineage, suggesting rapid global spread following emergence. This pattern contrasts with earlier A(H3N2) subclades that displayed more regionally constrained early expansion.

Subclade K (formerly designated J.2.4.1 and currently used in surveillance as part of the broader 2a.3a.1 genetic group) represents a recent diversification within seasonal influenza A(H3N2) viruses that has been associated with rapid international spread during the 2025/26 season. Molecular characterization in Oceania showed that subclade K viruses were genetically distinct from contemporaneous subclade J viruses and from the vaccine strain A/Croatia/10136RV/2023-like virus used in the 2025 southern hemisphere and 2025/26 northern hemisphere vaccines [6]. These observations align with the long-standing pattern of accelerated H3N2 evolution, in which substitutions and glycan remodeling on HA can rapidly alter antigenic properties while preserving receptor binding and replicative fitness [1,2].

At the HA level, subclade K viruses accumulated additional amino acid substitutions relative to earlier J.2.4 viruses (e.g., K2N, N158D, I160K, T328A, Q173R, S378N and S144N) [6]. This is relevant because the dominant neutralizing antibody response to H3N2 targets HA head epitopes close to the receptor-binding site; consequently, immune-driven drift frequently manifests as surface changes that reduce antibody binding while maintaining functional receptor engagement [1]. Within this framework, the S144N substitution is notable because it creates a potential N-linked glycosylation motif in the HA head [6]. Gain or loss of HA glycans is a recurrent mechanism of antigenic change in contemporary H3N2, and glycosylation differences can directly affect binding of antibodies elicited by egg-adapted vaccine strains [25]. Thus, the constellation of HA substitutions in subclade K is consistent with a lineage shaped by immune selection and constrained by receptor-binding requirements.

At the NA level, subclade K viruses formed a distinct NA clade characterized by a D346G substitution [6]. The co-occurrence of coordinated changes in both HA and NA is consistent with the concept of HA–NA functional balance, whereby alterations in HA receptor avidity and binding can be accompanied by compensatory NA changes to preserve efficient virion release and transmission [1]. More broadly, the rise of a successful lineage can reflect genome-wide compatibility and epistatic constraints across segments, including internal genes that modulate replication efficiency under immune pressure [5].

Antigenic characterization supported measurable divergence of subclade K in classical serology. In haemagglutination inhibition assays with post-infection ferret antisera, 204/205 (99.5%) of subclade K virus isolates tested in Australia showed a ≥8-fold reduction in reactivity to ferret antiserum raised against the cell-grown 2025 vaccine virus A/Croatia/10136RV/2023 [6]. Conversely, subclade K viruses showed better reactivity to antisera raised against selected J.2.4 viruses and to antiserum raised against a K virus (A/Darwin/1415/2025), indicating that the lineage was antigenically displaced from the immediate vaccine reference while remaining within the broader H3N2 antigenic space [6]. It is important to note that VE studies conducted in England, Canada, and China have reported moderate protection during the predominance of the K subclade, highlighting that reduced reactivity in ferret sera does not necessarily translate into a lack of protection at the population level [7,8,9,10].

### 3.2. Epidemiological Impact and Global Spread of Influenza A(H3N2) Subclade K

**Figure 2:**
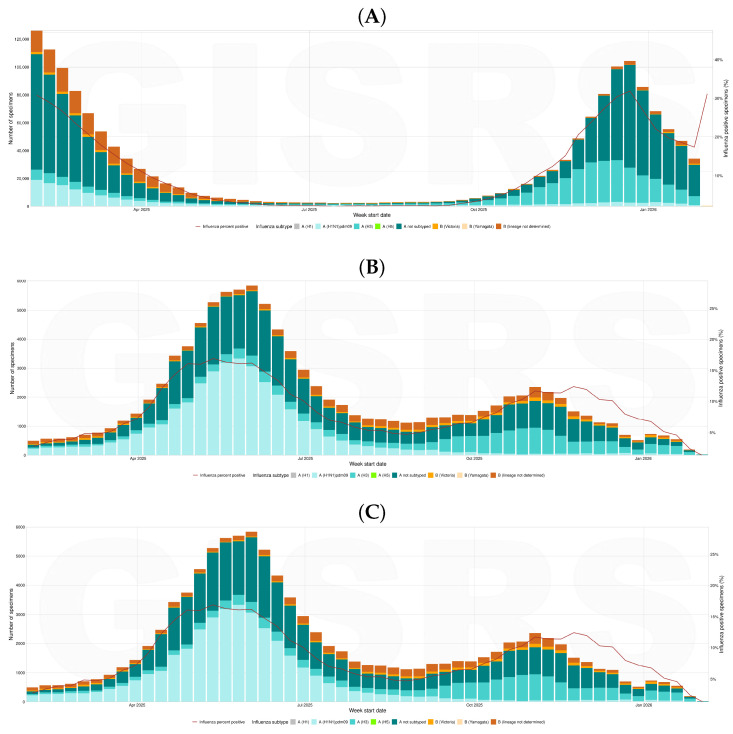
Global influenza A virus detections reported to the World Health Organization FluNet surveillance system from 6 February 2025 to 6 February 2026. Influenza detections are shown by subtype and surveillance reporting stream across three geographic aggregation categories available within the FluNet platform. Data were extracted using the FluNet interface with the surveillance site type filter set to “All”, which includes sentinel, non-sentinel, and reports where the surveillance stream is unspecified. Sentinel data represent systematic sampling from defined outpatient surveillance networks using standardized case definitions (e.g., influenza-like illness or acute respiratory infection). Non-sentinel data include detections reported outside sentinel systems, such as hospital-based diagnostic testing or outbreak investigations. Undefined stream data represent reports where the surveillance stream is not specified. Panels show aggregated detections reported to FluNet for (**A**) Northern Hemisphere temperate/sub-tropical regions, (**B**) Southern Hemisphere temperate/sub-tropical regions, and (**C**) tropical regions. These geographic categories follow the regional reporting framework used in the FluNet interface and should not be interpreted as strict climatological classifications. Countries with large geographic extent may include regions spanning multiple climate zones; however, surveillance data are reported at the national level and therefore contribute to the aggregated category defined by the reporting framework. Influenza A viruses reported without subtype designation are included as a separate category, reflecting routine surveillance outputs in which a proportion of influenza A detections are not further subtyped. Weekly counts represent aggregated detections and should be interpreted as indicators of temporal activity patterns rather than direct comparisons of incidence between regions, as testing intensity and reporting completeness may vary across countries.

The epidemiology of subclade K (J.2.4.1) has been characterized by rapid spread and early predominance within the wider 2a.3a.1 clade, with detections initially increasing in the southern hemisphere in 2025 and subsequently expanding to the northern hemisphere during the 2025/26 season, as shown in Figure 2.

In the Northern Hemisphere (Figure 2A), A(H3N2) showed a classic epidemic pattern: very low activity in spring–summer 2025, a sustained increase from autumn (from week 40 onward), and a sharp winter peak between late November 2025 and late January 2026. The highest weekly counts, reported mainly by non-sentinel sites, exceeded 11,000 cases, indicating a substantial hospital burden, while sentinel surveillance documented parallel community transmission with lower absolute numbers, as expected from targeted sampling. During peak weeks, A(H3N2) was the predominant influenza A subtype, clearly exceeding A(H1N1)pdm09 across all three surveillance streams.

In the Southern Hemisphere temperate/sub-tropical setting (Figure 2B), the seasonal pattern was broadly inverted, with limited activity in early 2025 followed by rapid growth during the austral autumn and a winter peak between May and July 2025; peak intensity was notably lower than in the Northern Hemisphere, with maxima on the order of hundreds of weekly detections in non-sentinel data.

In tropical regions (Figure 2C), A(H3N2) circulation was observable year-round with more irregular fluctuations and without a single sharp winter peak, with generally low weekly counts (often below 100), consistent with a more endemic transmission profile in which climatic drivers other than cold temperature may contribute to seasonality. At the global level, the WHO has reported increasing A(H3N2) activity with growing detections of subclade K in several regions, including South-East Asia [26]. In the Americas, PAHO noted that while widespread circulation had not yet been observed in South America, detections were increasing in North America [27]. In Europe and parts of Asia, multiple surveillance reports describe a marked rise of A(H3N2) viruses belonging to subclade K, without clear evidence of increased clinical severity to date [26,27].

Consistent patterns have been observed across major surveillance systems. In England, UKHSA genetic characterization data indicate that subclade K (J.2.4.1) rapidly became dominant, accounting for 97.1% (1297/1335) of characterized A(H3N2) viruses between week 40 2025 (week ending 5 October 2025) and week 3 2026 (week ending 18 January 2026) [28]. Similarly, in the United States, CDC FluView data show that 90.4% (596/659) of genetically characterized A(H3N2) viruses collected since week 40 2025 (28 September 2025) belonged to subclade K [29]. As of February 2026, ongoing surveillance updates from both regions continue to indicate sustained predominance of 2a.3a.1 viruses, with subclade K remaining the main contributor among characterized A(H3N2) detections, although proportions may vary over time as sequencing accrues. These findings suggest rapid expansion across multiple transmission networks rather than regionally confined spread.

## 4. Discussion

The genomic, antigenic, and epidemiological evidence presented in this study supports the interpretation that influenza A(H3N2) subclade K (J.2.4.1) represents a recently diversified lineage, characterized by coordinated evolution of the HA and NA surface glycoproteins and rapid international spread during the 2025/26 season. Our temporal phylogenetic analyses demonstrated concordant clustering of K-associated viruses in both gene segments, ruling out transient stochastic expansion and instead indicating sustained evolutionary success within the current H3N2 genetic background. The observed constellation of substitutions in HA at antigenically relevant sites fits within the established paradigm of progressive antigenic drift of H3N2, in which relatively limited sets of amino acid substitutions can significantly reduce antibody recognition while preserving receptor binding and viral fitness [30]. It is important to note that, although reduced reactivity in hemagglutination inhibition assays compared to vaccine reference antisera has been reported in several contexts, contemporary estimates of VE have remained moderate, highlighting the complex and often nonlinear relationship between antigenic distance measured in ferret serology and protection at the population level.

Considering this evolutionary and antigenic context, the constellation of substitutions observed in J.2.4.1 appears plausibly functional, suggesting advantages in terms of antigenic adaptation, receptor binding, and compatibility with NA activity and internal gene composition. In particular, this series of substitutions could (i) modify key antigenic surfaces on HA, (ii) preserve or modulate receptor interaction, and (iii) remain compatible with NA activity and internal gene constellations that support replication. It is important to note that rapid expansion of the subclade does not necessarily imply increased virulence; more likely, it reflects an increased ability to infect individuals with partial immunity or to trigger early transmission during the season.

The observed predominance of J.2.4.1 in the United Kingdom and the United States during the first half of the season aligns with the hypothesis of a transmission advantage that is detectable at the population level. Nevertheless, estimates of relative growth are sensitive to surveillance intensity, representativeness of sequenced specimens, and delays in reporting. Continued analyses using phylodynamic approaches with explicit sampling models, combined with antigenic cartography where available, will be helpful to quantify the magnitude of any intrinsic advantage and to separate biological effects from surveillance artifacts.

Our results are consistent with the broader narrative of ongoing antigenic drift in H3N2, in which relatively small sets of HA changes can meaningfully erode recognition by antibodies induced by prior infection or vaccination. This has practical implications for vaccine strain selection, particularly when candidate vaccine viruses are propagated in eggs, where additional adaptive substitutions may be selected and further distort antigenic match. This remains especially relevant for H3N2, as egg-adaptive changes near the receptor-binding site have been associated with reduced VE in some seasons. In this context, several studies have reported improved antigenic fidelity and, in some cases, higher effectiveness for cell-based or recombinant vaccines compared to egg-based formulations, including high-dose and adjuvanted vaccines, highlighting the impact of production platform on vaccine performance.

The emergence of J.2.4.1 further highlights that HA-only assessments may be insufficient to fully anticipate vaccine performance. NA antigenic drift and the breadth of NA-directed immunity can influence protection, particularly against severe disease, yet NA remains less standardized in vaccine composition and evaluation. Integrating NA sequence surveillance, neuraminidase inhibition data, and paired HA/NA evolutionary analyses should therefore be prioritized to better understand how combined antigenic trajectories shape population immunity and vaccine outcomes.

The evolutionary success of H3N2 lineages is often underpinned by coordinated changes across segments. Alterations in HA that affect receptor avidity can require compensatory changes in NA to maintain an optimal balance between attachment and release. Our paired analyses of HA and NA support the view that J.2.4.1 should be interpreted as a genome-wide phenotype rather than as an HA-only event. This has operational implications: tracking subclades using both HA and NA lineages, and evaluating reassortment signals when they occur, can improve the resolution of surveillance and the interpretation of antigenic data.

From a public health perspective, the rapid rise of J.2.4.1 reinforces the need for timely sharing of sequences and antigenic characterization data, particularly early in the season when vaccine strain decisions are being informed. In addition to routine genetic classification, near-real-time monitoring of substitutions at known antigenic sites, glycosylation motifs, and receptor-binding associated residues can provide early warning signals of potential antigenic change. Where feasible, coupling genomic surveillance with standardized antigenic assays and epidemiological indicators (e.g., age distribution, severity proxies, and vaccine breakthrough analyses) will help interpret whether emerging lineages are primarily immune-escape variants, transmission-enhanced variants, or both.

Given the global connectivity of influenza transmission, regional differences in timing and dominance should not be viewed in isolation. The apparent early signals in parts of the southern hemisphere, followed by widespread detections in the northern hemisphere, emphasize the importance of integrating datasets across regions. Investments in sequencing capacity, harmonized analytic pipelines, and rapid dissemination of interpretive reports will be crucial to reduce lag between viral evolution and public health action.

### 4.1. Limitations

This study has several limitations. First, the FluNet data used for epidemiological trend analysis are not standardized for population size, testing volume, or surveillance intensity. Substantial heterogeneity exists across countries in diagnostic capacity, healthcare access, and reporting completeness. Consequently, aggregated counts may be disproportionately influenced by nations with higher testing volumes and more comprehensive surveillance systems, potentially biasing the observed temporal and geographic patterns. Furthermore, the use of predefined FluNet geographic categories—based on operational reporting groupings rather than strict climatological definitions—may introduce classification ambiguity. This is particularly relevant for large countries spanning multiple climatic zones, where national-level aggregation may obscure important subnational variation in transmission dynamics. Additionally, a proportion of influenza A detections are reported without subtype designation; these may not be randomly distributed across time or geography, reflecting differences in laboratory capacity or testing priorities, which may affect the accuracy of subtype-specific trend interpretation. Second, the representativeness of publicly available sequences from GISAID may vary substantially by country, time period, and sampling frame, potentially biasing estimates of lineage frequency and geographic spread. Third, antigenic characterization data are not uniformly available across regions and may be influenced by assay choice, virus isolation history, and laboratory-specific protocols. Fourth, phylogenetic inference is sensitive to sequence quality, alignment choices, and model assumptions; although our analyses employed standard, reproducible approaches, alternative models might yield modestly different estimates of timing and branching patterns. Finally, we did not directly estimate VE or clinical severity associated with subclade K (J.2.4.1); therefore, our conclusions focus on evolutionary dynamics and antigenic signals rather than on clinical impact.

### 4.2. Conclusions

In summary, our molecular characterization of influenza A(H3N2) subclade K (J.2.4.1) indicates that its rapid rise during the 2025/26 season is consistent with successful dissemination accompanied by antigenic drift relative to contemporary vaccine reference strains. From an epidemiological perspective, this rapid predominance is compatible with a modest but meaningful transmission advantage, potentially driven by improved fitness under existing population immunity, enhanced seeding during early-season travel, or favorable antigenic positioning relative to prior vaccine strains. However, increased prevalence does not necessarily imply increased virulence, as current public health reports primarily highlight expanded detection and early-season activity rather than a clear signal of increased clinical severity. These observations underscore the persistent challenges posed by H3N2 for vaccine strain selection and highlight the value of integrating HA/NA genomic surveillance with antigenic and epidemiological data streams. Continued monitoring of J.2.4.1 and its descendants will be essential to determine whether its predominance persists, whether further antigenic drift accumulates, and how these changes should inform next-season vaccine composition and preparedness planning. From a public health perspective, these findings reinforce the need for sustained and globally representative genomic surveillance with timely sequence sharing and adequate geographic coverage to support early detection of emerging variants and improve pandemic preparedness.

## Data Availability

Aggregated epidemiological time-series data were obtained from the WHO FluNet platform (https://www.who.int/tools/flunet, accessed on 10 February 2026). Influenza A(H3N2) HA and NA sequences were downloaded from GISAID (accessed on 10 February 2026), subject to GISAID’s terms of use.

## References

[1] Allen J.D., Ross T.M. (2018). H3N2 influenza viruses in humans: Viral mechanisms, evolution, and evaluation. Hum. Vaccines Immunother..

[2] Yang H., Carney P.J., Chang J.C., Guo Z., Villanueva J.M., Stevens J. (2015). Structure and receptor binding preferences of recombinant human A(H3N2) virus hemagglutinins. Virology.

[3] Wu N.C., Lv H., Thompson A.J., Wu D.C., Ng W.W.S., Kadam R.U., Lin C.W., Nycholat C.M., McBride R., Liang W. (2019). Preventing an Antigenically Disruptive Mutation in Egg-Based H3N2 Seasonal Influenza Vaccines by Mutational Incompatibility. Cell Host Microbe.

[4] Lei H., Xiao B., Lin X., Liang Z., Ling S., Bai Y., Dhanasekaran V., Song W., Wong S.S., Zanin M. (2025). Chronology of H3N2 human influenza virus surface glycoprotein adaptation from 1968 to 2019 reveals a surge of adaptation between 1997 and 2002. J. Virol..

[5] Vigeveno R.M., Han A.X., de Vries R.P., Parker E., de Haan K., van Leeuwen S., Hulme K.D., Lauring A.S., Te Velthuis A.J., Boons G.J. (2024). Long-term evolution of human seasonal influenza virus A (H3N2) is associated with an increase in polymerase complex activity. Virus Evol..

[6] Dapat C., Peck H., Jelley L., Diefenbach-Elstob T., Slater T., Hussain S., Britton P., Cheng A.C., Wood T., Howard-Jones A. (2025). Extended influenza seasons in Australia and New Zealand in 2025 due to the emergence of influenza A(H3N2) subclade K viruses. Eurosurveillance.

[7] Kirsebom F.C., Thompson C., Talts T., Kele B., Whitaker H.J., Andrews N., Aziz N.A., Rawlinson C., Green R.E., Quinot C. (2025). Early influenza virus characterisation and vaccine effectiveness in England in autumn 2025, a period dominated by influenza A (H3N2) subclade K. Eurosurveillance.

[8] Zambon M., Hayden F.G. (2026). Influenza A(H3N2) Subclade K Virus: Threat and Response. JAMA.

[9] Separovic L., Sabaiduc S., Zhan Y., Kaweski S.E., Olsha R., Hasso M., Mather R.G., Carazo S., Lacroix C., Meunier I. (2026). Interim 2025/26 influenza vaccine effectiveness estimates with immuno-epidemiological considerations for A(H3N2) subclade K protection, Canada, January 2026. Eurosurveillance.

[10] Shen Y., Zhang D., Feng Z., Ma C., Shi W., Duan W., Li J., Zhang L., Wu D., Zhang J. (2026). Moderate protection from vaccination against influenza A(H3N2) subclade K in Beijing, China, September to December 2025. Eurosurveillance.

[11] World Health Organization Recommended Composition of Influenza Virus Vaccines for Use in the 2025 Southern Hemisphere Influenza Season, 2024. Technical Document (27 September 2024). https://www.who.int/publications/m/item/recommended-composition-of-influenza-virus-vaccines-for-use-in-the-2025-southern-hemisphere-influenza-season.

[12] World Health Organization Recommended Composition of Influenza Virus Vaccines for Use in the 2025–2026 Northern Hemisphere Influenza Season, 2025. Technical Document (28 February 2025). https://iris.who.int/server/api/core/bitstreams/fc765188-9f02-4f81-b487-88696b4f3010/content.

[13] Centers for Disease Control and Prevention Influenza Activity in the United States During the 2024–25 Season and Composition of the 2025–26 Influenza Vaccine, 2025. CDC Influenza Update. https://www.cdc.gov/flu/whats-new/2025-2026-influenza-activity.html.

[14] U.S. Food and Drug Administration Influenza Vaccine Composition for the 2025–2026 U.S. Influenza Season, 2025. Vaccines, Blood & Biologics. https://www.cdc.gov/flu/season/2025-2026.html.

[15] European Medicines Agency EU Recommendations for 2025/2026 Seasonal Flu Vaccine Composition, 2025. News Item (Updated 9 April 2025). https://www.ema.europa.eu/en/news/eu-recommendations-2025-2026-seasonal-flu-vaccine-composition.

[16] European Centre for Disease Prevention and Control Influenza Virus Characteristics, Week 40 2024 to Week 33 2025, EU/EEA, 2025. ECDC Report. https://www.ecdc.europa.eu/en/publications-data/influenza-virus-characteristics-week-40-2024-week-33-2025.

[17] World Health Organization Weekly Epidemiological Record, 2026. https://iris.who.int/handle/10665/385047.

[18] Neher R.A., Huddleston J., Bedford T., Lewis N.S., Harvey R., Galiano M., Byrne A.M., James S., Smith D., Łuksza M. (2026). Nomenclature for tracking of genetic variation of seasonal influenza viruses. Influenza Other Respir. Viruses.

[19] Li H. (2018). Minimap2: Pairwise alignment for nucleotide sequences. Bioinformatics.

[20] Moshiri N. (2021). ViralMSA: Massively scalable reference-guided multiple sequence alignment of viral genomes. Bioinformatics.

[21] Larsson A. (2014). AliView: A fast and lightweight alignment viewer and editor for large datasets. Bioinformatics.

[22] Minh B.Q., Schmidt H.A., Chernomor O., Schrempf D., Woodhams M.D., Von Haeseler A., Lanfear R. (2020). IQ-TREE 2: New models and efficient methods for phylogenetic inference in the genomic era. Mol. Biol. Evol..

[23] Sagulenko P., Puller V., Neher R.A. (2018). TreeTime: Maximum-likelihood phylodynamic analysis. Virus Evol..

[24] Xu X., Hull W., Plunkett D., Tu Z.J., Ross T.M., Rhoads D.D., Wang H. (2026). Emergence of influenza A(H3N2) subclade K in northeast Ohio in autumn 2025. J. Clin. Microbiol..

[25] Zost S.J., Parkhouse K., Gumina M.E., Kim K., Diaz Perez S., Wilson P.C., Treanor J.J., Sant A.J., Cobey S., Hensley S.E. (2017). Contemporary H3N2 influenza viruses have a glycosylation site that alters binding of antibodies elicited by egg-adapted vaccine strains. Proc. Natl. Acad. Sci. USA.

[26] World Health Organization Seasonal Influenza—Global Situation, 2025. Disease Outbreak News (DON586). https://www.who.int/emergencies/disease-outbreak-news/item/2025-DON586.

[27] Pan American Health Organization (2025). PAHO Calls for Strengthened Vaccination and Surveillance in the Americas Amid a Global Increase of Influenza A(H3N2) Subclade K, 2025. PAHO/WHO News Release.

[28] UK Health Security Agency National Flu and COVID-19 Surveillance Report: 22 January 2026 (Week 4), 2026. GOV.UK. https://www.gov.uk/government/statistics/national-flu-and-covid-19-surveillance-reports-2025-to-2026-season/national-flu-and-covid-19-surveillance-report-22-january-2026-week-4.

[29] Centers for Disease Control and Prevention Weekly US Influenza Surveillance Report: Key Updates for Week 2, Ending January 17, 2026. FluView. https://www.cdc.gov/fluview/surveillance/2026-week-02.html.

[30] Villa M., Lässig M. (2017). Fitness cost of reassortment in human influenza. PLoS Pathog..

